# The ethylene response factor *AtERF4* negatively regulates the iron deficiency response in *Arabidopsis thaliana*

**DOI:** 10.1371/journal.pone.0186580

**Published:** 2017-10-18

**Authors:** Wei Liu, N. J. Umuhoza Karemera, Ting Wu, Yafei Yang, Xinzhong Zhang, Xuefeng Xu, Yi Wang, Zhenhai Han

**Affiliations:** 1 Institute for Horticultural Plants, College of Horticulture, China Agricultural University, Beijing, China; 2 Key Laboratory of Beijing Municipality of Stress Physiology and Molecular Biology of Fruit Trees, Beijing, China; National Taiwan University, TAIWAN

## Abstract

Iron (Fe) deficiency is one of many conditions that can seriously damage crops. Low levels of photosynthesis can lead to the degradation of chlorophyll content and impaired respiration in affected plants, which together cause poor growth and reduce quality. Although ethylene plays an important role in responses to Fe deficiency, a limited number of studies have been carried out on ethylene response factor (ERFs) as components of plant regulation mechanisms. Thus, this study aimed to investigate the role of *AtERF4* in plant responses to Fe deficiency. Results collected when *Arabidopsis thaliana* was grown under Fe deficient conditions as well as in the presence of 1-aminocyclopropane-1-carboxylic acid (ACC) revealed that leaf chlorosis did not occur over short timescales and that chloroplast structural integrity was retained. At the same time, expression of the chlorophyll degradation-related genes *AtPAO* and *AtCLH1* was inhibited and net H^+^ root flux was amplified. Our results show that chlorophyll content was enhanced in the mutant *erf4*, while expression of the chlorophyll degradation gene *AtCLH1* was reduced. Ferric reductase activity in roots was also significantly higher in the mutant than in wild type plants, while *erf4* caused high levels of expression of the genes *AtIRT1* and *AtHA2* under Fe deficient conditions. We also utilized yeast one-hybrid technology in this study to determine that *AtERF4* binds directly to the *AtCLH1* and *AtITR1* promoter. Observations show that transient over-expression of *AtERF4* resulted in rapid chlorophyll degradation in the leaves of *Nicotiana tabacum* and the up-regulation of gene *AtCLH1* expression. In summary, AtERF4 plays an important role as a negative regulator of Fe deficiency responses, we hypothesize that *AtERF4* may exert a balancing effect on plants subject to nutrition stress.

## Introduction

Iron (Fe) is one of the most important micronutrients for most living organisms; in plants, this element plays a key role in many critical processes throughout life [1]. However, because the solubility of Fe is very low in alkaline or calcified soils, the potential for a plant to uptake this element can often be very limited. The adaptations for Fe absorption used by higher plants have been grouped into reduction (strategy I) and Fe-chelating mechanisms (strategy II) [1, 2]. Mechanism I is found in dicotyledonous plants because soil acidification increases the solubility of Fe in rhizosphere particles via the exchange of Fe^3+^ to Fe^2+^ through reduction of the former; an Fe (II) transporter then moves Fe^2+^ across the plasma membrane into cells [3]. Research on how plant Fe deficiency responses are regulated has become increasingly focused as the functional genes involved in this process (e.g. *IRT*, *FRO* [4, 5]) have been revealed. The factors necessary for transcriptional regulation, FIT and its functional homologue FER [6, 7], as well as numerous members of the bHLH family (e.g. bHLH38, bHLH39, bHLH100, bHLH101, and bHLH068) [8–12], have been studied in detail in order to elucidate their roles in plant Fe deficiency responses. It is known, for example, that ILR3 and bHLH115 (both members of the *Arabidopsis* bHLH transcription factor family) interact with PYE [13], while bHLH104 interacts with ILR3, and both regulate the expression of Ib subgroup bHLH genes and PYE by binding to their promoters and increasing plant tolerance to low Fe concentrations [14]. At the same time, MdbHLH104 directly regulates the expression of *MdbHLH38*, *MdbHLH39*, and *MdbPYE* to control apple tree Fe uptake (i.e. *Malus* × *domestic* cv. ‘Royal Gala’) [15].

A number of hormones and their analogues, including auxin (IAA) [16], cytokinins (CKs) [17], jasmonic (JA) [18] and abscisic acid (ABA) [19], nitric oxide (NO) [20–22] and ethylene (ET) [23, 24], are thought to participate in plant responses to Fe deficiency. Ethylene is one of the five main plant hormones; because this hydrocarbon regulates RNA and protein synthesis, it is often involved in plant growth, development, and stress resistance processes. This hormone act as a signal substance that mitigates abiotic stress in response to drought [25] and heavy metal stresses [26].

A number of noteworthy aspects of Fe deficiency response regulation involve an ethylene signal. For example, Fe deficiency can induce the synthesis of ethylene in strategy I plants, and this hormone also positively regulates responses to low levels of this element [21, 23, 27, 28]. The ethylene content in cucumber and pumpkin roots was significantly increases under conditions of low iron stress [23, 24], for example, a process which contributes to the balance between of Fe and K, while an increase in ethylene content given an Fe deficiency also contributes to the growth of principal roots in *Arabidopsis* [29]. It has also been reported that rice treated for five days with 1-aminocyclopropane-1-carboxylic acid (ACC) experienced significant up-regulation of the Fe absorption and transport-related genes *OsIRT1*, *OsIRO2*, and *OsNAS1* [30].

To a large extent, the impact of ethylene on Fe deficiency responses reflects the regulation of downstream transcription factors. These primarily include the ETHYLENE INSENSITIVE3 and ETHYLENE INSENSITIVE3-LIKE1 groups (*EIN3*/*EILI*) as well as ethylene response factors (*ERFs*) [31, 32]. Ethylene signals to *EIN3/EIL1* and then regulates the expression of an ERF transcription factor which ultimately induces a series of ethylene-responsive genes [33]. Lingam et al. [34] studied the molecular mechanism underlying the interaction between ethylene signals and Fe deficiency; this signaling also participates in the prevention of photooxidation and seedling chlorosis, as *EIN3/EIL1* directly activate their target chlorophyll biosynthesis genes, *PORA* and *PORB*, which encode the original chlorophyll ester oxide reductase) [35]. Thus, although ethylene production under stress conditions can regulate the expression of chlorophyll biosynthesis genes through *EIN3/EIL1* to enhance photosynthesis [36], few studies have determined the roles of *ERF* in the Fe deficiency response regulation mechanisms of plants.

ERFs comprise a subfamily of the AP2/ERF transcription factor family, which possesses an AP2 domain at the protein N terminal composed of 58 amino acids. There are 65 members of the ERF subfamily [37]; these factors have been studied in many plants, including *Arabidopsis*, rice, tomato, tobacco, alfalfa, soybeans, and barley, largely because of the prominent roles they play in the regulation of plant biotic and abiotic stress responses [38]. Members of the ERF subfamily function as components of the innate immune mechanisms and signaling pathways of disease resistance [39, 40], and in plant tolerance to abiotic stresses, such as low temperature, drought, and low oxygen, as well as highly osmotic and saline conditions [41–44]. Some ERF transcription factors are also capable of inhibiting transcription, which has an adverse effect on the expression of stress-related genes; for example, AtERF7 in *Arabidopsis* binds to the GCC-box domain and inhibits other ERF transcription factors [45].

AtERF4 is a negative regulatory protein that is involved in ET and ABA responses; alongside JA, these hormones induce *AtERF4* expression [46]. Research has shown that tobacco plants containing transgenic *GmERF4* exhibit a higher tolerance to salt and drought stress than their wild-type counterparts [43]. This co-expression shows that *AtERF4* is also important in Fe deficiency stress responses [47].

ERF transcription factors are also involved in chlorophyll degradation; in citrus, for example, *CitERF13* binds directly to the chlorophyll degradation gene *CitPPH* promoter and regulates this process during fruit ripening [48]. The most distinct characteristic of an Fe deficient plant phenotype is interveinal chlorosis; over short timescales, Fe deficiency also reduces the pH of the rhizosphere and increases the number of root hairs, while changes in *ERF4* expression also effects salt and Fe stress [32, 49]. We therefore hypothesize that *ERF4* may play a significant role in response to Fe deficiency. The results of this study show that an absence of *ERF4* increases the Fe deficiency response, while *AtERF4* regulates the expression of both Fe uptake and chlorophyll degradation genes via a yeast one-hybrid. We therefore propose a negative regulatory pathway for these classic phenomena.

## Materials and methods

### Plant material and growth conditions

Seeds of *Arabidopsis thaliana* Col-0 wild type and T-DNA insertion lines for *erf4* (SALK_073394C) were purchased for this study from the TAIR website (https://www.arabidopsis.org/). The *erf4* mutant was screened using PCR using T-DNA (LBb1.3) and gene-specific primers (S1 Table), and all seeds were washed and vernalized prior to germination. Seedlings of uniform size were then chosen from plates containing 0.75% agar-solidified Murashige and Skoog (MS) nutrient medium and adjusted to pH 5.8 with 1 M NaOH two weeks after sowing. All plants were grown at 24°C under a light intensity of 200 μmol photons m^−2^ s^−1^ and a 16 h light / 8 h dark rhythm. Some plants were transferred to MS solid medium (100 μM Fe, as a control), while Fe deficient (0 μM Fe, the complete nutrient solid medium, but without Fe) treatments also incorporated additions of either 10 μM ACC or 10 μM aminoethoxyvinylglycine (AVG) on the basis of different Fe concentration respectively. Roots and leaves were harvested at time zero and after two days of treatments.

### Measurement of Fe and magnesium (Mg) content

Seedling roots and shoots were washed with water and wiped dry before being transferred to 65°C conditions [11]. Subsequent to grinding, 0.1g of powder was added to 5 mL HNO_3_ static for 30 minutes, and samples were put in a microwave digestion instrument at 180°C for 25 minutes to enable volume determination at 25 mL. Both Fe and Mg contents were determined using a plasma spectrum analyzer (ICP) (OPTIMA3300DV, Perkin Elmer, Shelton, CT, USA). Each treatment measurement was performed using three biological replicates.

### Measurements of net H^+^ flux using non-invasive micro-test technology (NMT)

We measured net H^+^ flux using a non-invasive scanning ion-selective electrode technique (Xuyue Sci. & Tech. Co., Beijing; www.xuyue.net). Thus, H^+^ selective microelectrodes were deployed for detection within the root hair zone, and samples were tested in pH 6.0 solution. The test liquid comprised 0.1 mM MgCl_2_, 0.1 mM KCl, 0.1 mM CaCl_2_, 0.2 mM Na_2_SO_4_, 0.3 mM MES, and 0.5 mM NaCl, while pH 6.0 was maintained using choline and HCl [50]. Electrodes with Nernstian slope > 50 mV per decade were used, and H^+^ fluxes were calculated using the free MS Excel spreadsheet JCal V3.3 (http://www.youngerusa.com or http://www.xuyue.net). Ion flux was calculated using Fick’s law of diffusion, as follows:

$$J=-D_{0}\times \frac{dc}{dx}.$$

In this expression, *J* denotes ion flux, *D*_*0*_ is the diffusion constant, *dc* is the ion concentration gradient, and *dx* is the distance between two points. The direction of flux was then derived using Fick’s law of diffusion.

### Scanning electron microscope (SEM) and transmission electron microscope (TEM) observations

We analyzed root hair patterns using an SEM. In this step, excised root samples of *A*. *thaliana* wild type and *erf4* were immediately soaked in 2.5% glutaraldehyde and 1% osmic acid, fixed for two hours, and then placed in a 0.1 M phosphate buffer and washed three times. Fixed samples were then dehydrated through an ethanol series and dried with a critical point drying apparatus (EM CPD, LEICA, Germany). These dried samples were then mounted on aluminum stubs, coated with a thin layer of gold film in a sputter coater (IB-3, EIKO, Japan), and observed with a SEM (S-3400N, Hitachi, Japan).

We observed chloroplast ultrastructure using a TEM. In this step, freshly excised leaves of wild type and *erf4* plants were submerged in 2.5% glutaraldehyde and 1% osmic acid, fixed for two hours, and then washed three times in a 0.1 M phosphate buffer. Fixed samples were then dehydrated using an acetone series and embedded with epoxy resin before being slicing with an ultra microtome (EM UC6, LEICA, Germany), stained using uranyl acetate and lead citrate solution, and imaged using a TEM (JEM-1230, JEOL, Japan).

### Chlorophyll content measurements

We placed plant leaves in 80% acetone for 24 hours and detected the absorbance values of A_645_ and A_663_ using a spectrophotometer [51]. We used the chlorophyll content formula, as follows:

$$(\text{mg}/\text{g})=\frac{(20.3\times {\text{A}}_{645}+8.04\times {\text{A}}_{663})\times \text{V}}{\text{W}\times 10^{3}}.$$

In this expression, V denotes extract volume (mL), while W is the weight (g) of fresh leaves. This experiment was repeated three times for each group, and the total chlorophyll content (SPAD value) of leaves was measured using a portable chlorophyll meter (SPAD-502 Plus, KONICA, Japan).

### Detection of rhizosphere pH location

Plant roots was embedded in a culture medium containing 0.7% agar, 0.05 mM CaSO4, and 0.005% of the pH indicator bromocresol purple for 24 hours before they were observed and photographed [52].

### Measurement of Fe^3+^ reductase activity

Roots of *A*. *thaliana* were soaked in saturated solution CaSO_4_ for five minutes before they were cleaned with a solution of distilled water and transferred to the culture solution (100 μM Fe (III)-NaEDTA, 400 μM 2,2-bipyridyl) for one hour in the dark. The liquid absorbance reaction was then detected at A_520_ to calculate ferric reductase (FCR) activity as follows:

$$\text{FCR}\;(\text{nmol}\cdot {\text{g}}^{-1}\cdot {\text{h}}^{-1})=\frac{\text{V}\times {Α}_{520}\times 10^{9}}{8650\times \text{M}\times \text{T}}.$$

In this expression, V is the reaction solution volume (L), M is root weight (g), and T is reaction time (h). This experiment was performed in triplicate biological replicates in each group.

### Isolation of total RNA and quantitative real-time PCR

We extracted leaf and root total RNA using a TRIzol reagent (Invitrogen). Thus, utilizing DNaseI (Biotechnology Co., Ltd., Dalian, China) treatment, 2 μg of total RNA was then used for reverse transcription and gene relative expression levels were detected by using an Applied Biosystem 7500 Real-time PCR System. The *Arabidopsis Actin2* gene was used as the reference for this step; the primers (F) 5′-GGTAACATTGTGCTCAGTGGTGG-3′ and (R) 5′-AACGACCTTAATCTTCATGCTGC-3′ were designed using the software Primer Premier 5 (Premier Biosoft, USA) [53]. Relative expression was calculated using the 2^−ΔΔCT^ method [54]; a complete list of primers is presented in S1 Table. Each reaction was performed in triplicate biological replicates.

### Yeast one-hybrid assays

We performed a yeast one-hybrid assay as described by Lin et al. [55]. Briefly, coding sequence (CDS) of *AtERF4* were cloned into the EcoR I and Xho I sites of the pJG4-5 vector to form a GAD AtERF4 construct which was then cotransformed with various *LacZ* reporter gene constructs into the yeast strain EGY48 as outlined in the Yeast Protocols Handbook (Clontech). Transformants were then grown for two-to-three days on a minimal medium/-Ura,-Trp containing 5-bromo-4-chloro-3-indolyl-β-D- galactopyranoside (X-gal) in order to observe the color development of yeast colonies. Primers used for vector construction are presented in S1 Table.

### Transient expression analysis in plant

The CDS of *AtERF4* was cloned into the Xba I and BamH I sites of the pBI121 vector to form a pBI121-*AtERF4* construct which was then infiltrated in tobacco (*Nicotiana tabacum*) leaves. These plants were then grown for seven days following infiltration before transformed specimens were collected for further gene expression analysis.

*Arabidopsis thaliana* transient expression assay was performed as described by Dai et al. [56], with some modifications. *A*. *thaliana* seedlings were collected after one month of growth in growth chamber, and vacuum infiltration was performed under a pressure at 0.7 MPa. After release of the vacuum, seedlings were washed in deionized water and kept in deionized water for 24 hours at 8°C, followed by cultivation in MS nutrition solution. Plants were grown under conditions of 24°C, a light intensity of 200 μmol photons m^−2^ s^−1^ and 16-h light/ 8-h dark rhythm. After 5 days, transformed plant roots were used for RNA isolation, which was thereafter used for further gene expression analysis.

### Chlorophyll fluorescence measurements

Non-invasive chlorophyll fluorescence (i.e. maximal quantum efficiency of PSII, Fv/Fm) was measured at room temperature using an photosynthesis yield analyzer (MINI-PAM-II, WALZ, Germany) after dark-adapting plants for 20 minutes [57].

### Statistical analyses

All statistical analysis was performed using the SPSS 13.0 program (IBM Co, Armonk, USA), including the use of one-way analysis of variance (ANOVA) and Duncan’s multiple-range test.

## Results

### Ethylene is involved in Fe deficiency responses

**Fig 1:**
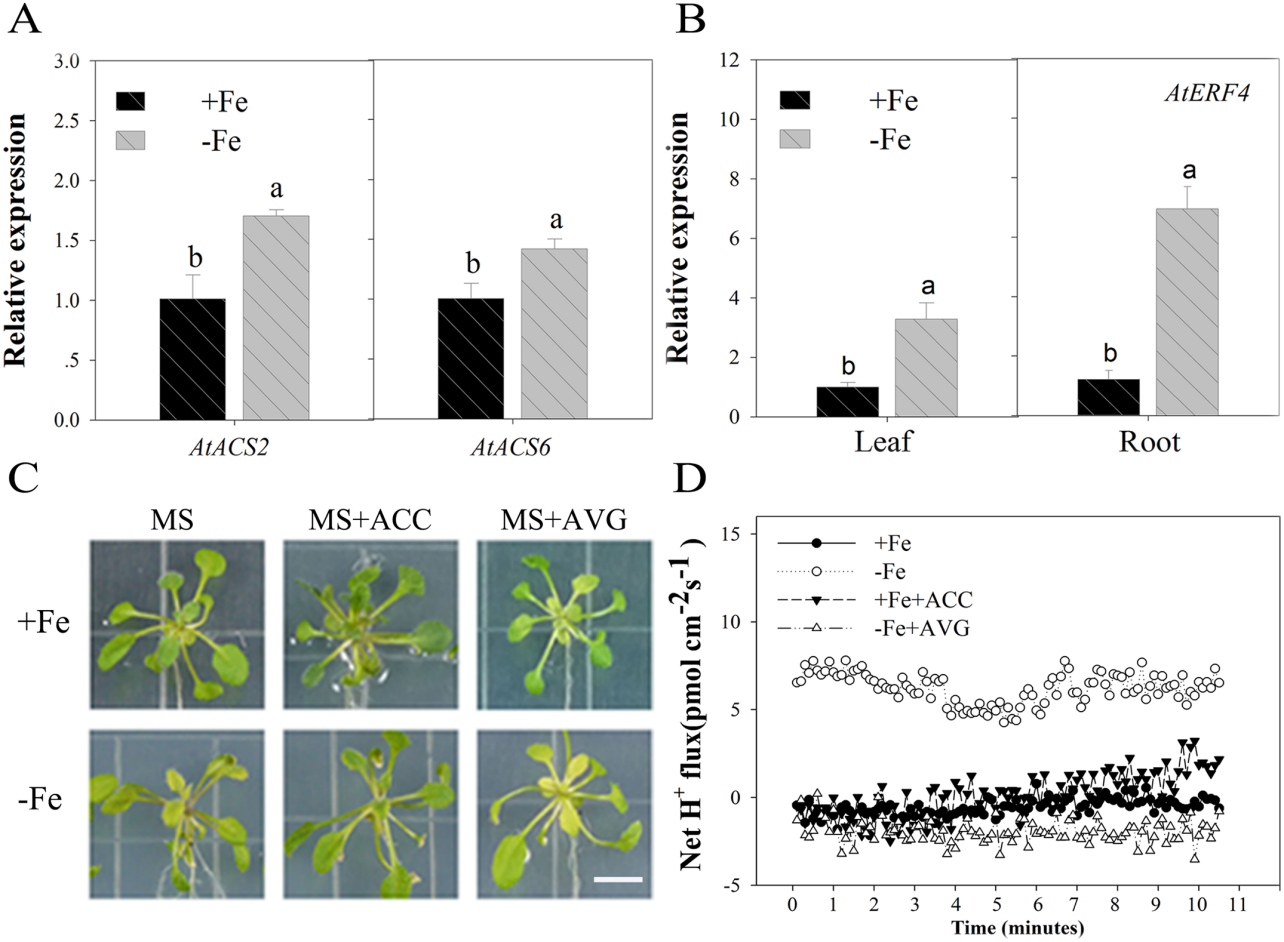
The phenotype of wild-type *A*. *thaliana* treated with Fe deficiency and exogenous ethylene. (A) Transcript abundances of the *ACS2* and *ACS6* genes in wild-type *Arabidopsis* under Fe-sufficient (100 μM EDTA-Fe) and Fe-deficient (0 μM EDTA-Fe) conditions for 2 d. Bars represent means ± SE of three replicates. Different letters represent statistically different means at *P* < 0.05 (one-way ANOVA with a Duncan post-hoc test). (B) Analysis of tissue expression of the ethylene response factor *AtERF4* under Fe-sufficient and Fe-deficient conditions for 2 d. Bars represent means ± SE of three replicates. Different letters represent statistically different means at *P* < 0.05 (one-way ANOVA with a Duncan post-hoc test). (C) The phenotype of the wild type with the exogenous ethylene promoter ACC or the ethylene inhibitor AVG treated for 5 d, scale bar = 0.8 cm. (D) The H^+^ flux of the wild-type root hair area with the exogenous ethylene promoter ACC or the ethylene inhibitor AVG treated for 5 d.

Previous research has shown that ethylene is produced for two days under conditions of Fe deficiency in *A*. *thaliana* [58] and some authors have noted that *ERF4* may have an influence on the Fe deficiency response [32, 47]. However, the mechanisms underlying how *AtERF4* regulates Fe deficiency responses has not yet been investigated. We therefore induced the expression of *AtACS2* and *AtACS6* in roots and leaves of wild-type plants via Fe deficiency stress for two days (Fig 1A); the results of this experiment showed that the amount of ethylene synthesized increased in the early stages of Fe deficiency and that the *AtERF4* gene was up-regulated in roots and leaves for two days because of this stress (Fig 1B).

Thus, to determine whether, or not, induced ethylene is involved in responses to Fe deficiency, we added the ethylene precursor ACC and the ethylene synthesis inhibitor AVG [59] to our growth media. Results show that the leaves of Fe deficient wild type plants exhibited symptoms of chlorosis, as expected; however, treatment with ACC alleviated the leaf chlorosis phenomena caused by Fe deficiency (i.e. Fe 100 μM with ACC and Fe 0 μM with ACC; Fig 1C). Previous research has demonstrated that ethylene has an effect on the acidification capacity of the root system [28, 60]; in this study, we also found that treatment with AVG effectively reduces H^+^ efflux velocity in wild type plants (Fig 1D). This result shows that ethylene plays an important role in releasing H^+^ as one component of the Fe deficiency response system. Given a normal supply of Fe (i.e. Fe 100 μM), addition of ACC increased H^+^ efflux to a certain extent, but much lower than seen under Fe deficient conditions.

Our results indicate that when plants suffer from Fe deficiency stress, they may respond by producing ethylene to activate regulatory factors that help increase the hydrogen capacity of the root system. This ultimately changes local environmental acidification and facilitates Fe absorption.

### Ethylene affects chlorophyll content in the early stages of Fe deficiency

**Fig 2:**
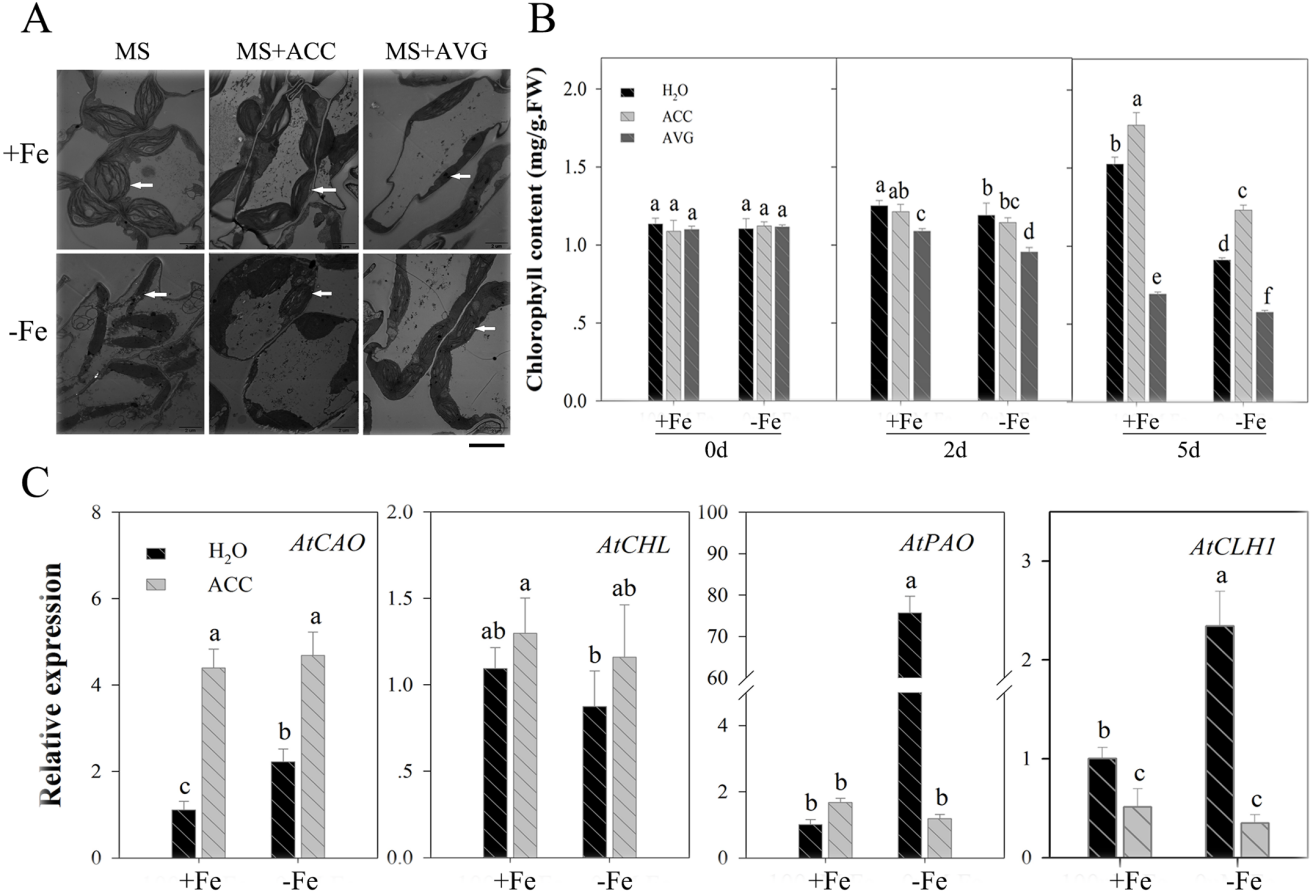
Effect of the exogenous ethylene promoter ACC or the ethylene inhibitor AVG treatment on chlorophyll content and gene expression in wild-type *A*. *thaliana*. (A) The chloroplast structure of the wild type (WT) in the treatment with the exogenous ethylene promoter ACC and the ethylene inhibitor AVG for 5 d was investigated by transmission electronic microscopy. White arrows indicate the chloroplast structure state of the seedling leaves, scale bar = 3 μm. (B) The chlorophyll content of WT in the treatment of the exogenous ethylene promoter ACC and the ethylene inhibitor AVG for 0, 2, and 5 d. Bars represent means ± SE of three replicates. Different letters represent statistically different means at *P* < 0.05 (one-way ANOVA with a Duncan post-hoc test). (C) Expression of the chlorophyll biosynthesis gene (*AtCAO*, *AtCHL*) and degradation gene (*AtPAO*, *AtCLH1*) of WT in the treatment of exogenous ethylene promoter ACC for 2 d. Bars represent means ± SE of three replicates. Different letters represent statistically different means at *P* < 0.05 (one-way ANOVA with a Duncan post-hoc test).

We investigated chloroplast structure using TEM (Fig 2A). These observations show that the external application of ethylene inhibitors (AVG) promotes chloroplast degradation. Thus, the chloroplast structure of leaves was partly damaged following AVG treatment for five days such that photosynthetic electron transfer chain functions no longer operated. Observations show that changes to chloroplast structures were more complete in Fe-deficient plants following ACC application than when this treatment was not applied. Ethylene therefore assists in stabilizing the structure of the chloroplast to a certain extent by delaying the chlorophyll degradation caused by Fe deficiency. Results show that the ethylene inhibitor, AVG, significantly decreased chlorophyll content (Fig 2B); the contents of this molecular reached its lowest value with AVG after five days of treatment. The opposite effect was seen following ACC treatment.

We also analyzed the expression of chlorophyll synthesis and degradation-related genes in this study (Fig 2C). Results show that transcription levels of the chlorophyll synthesis gene *AtCAO* were up-regulated by ACC, while those of the chlorophyll degradation genes *AtPAO* and *AtCLH1* were also up-regulated in Fe deficient plants; at the same time, however, ACC significantly decreased the expression of *AtPAO* and *AtCLH1*. These results show that ethylene production in the early stages of Fe deficiency exerts a discernible influence on chlorophyll degradation mitigation. It is possible that this is due to the fact that both chlorophyll synthesis and degradation processes are affected by the regulation of Fe absorption and transport.

### Mutant *erf4* Fe deficiency response

**Fig 3:**
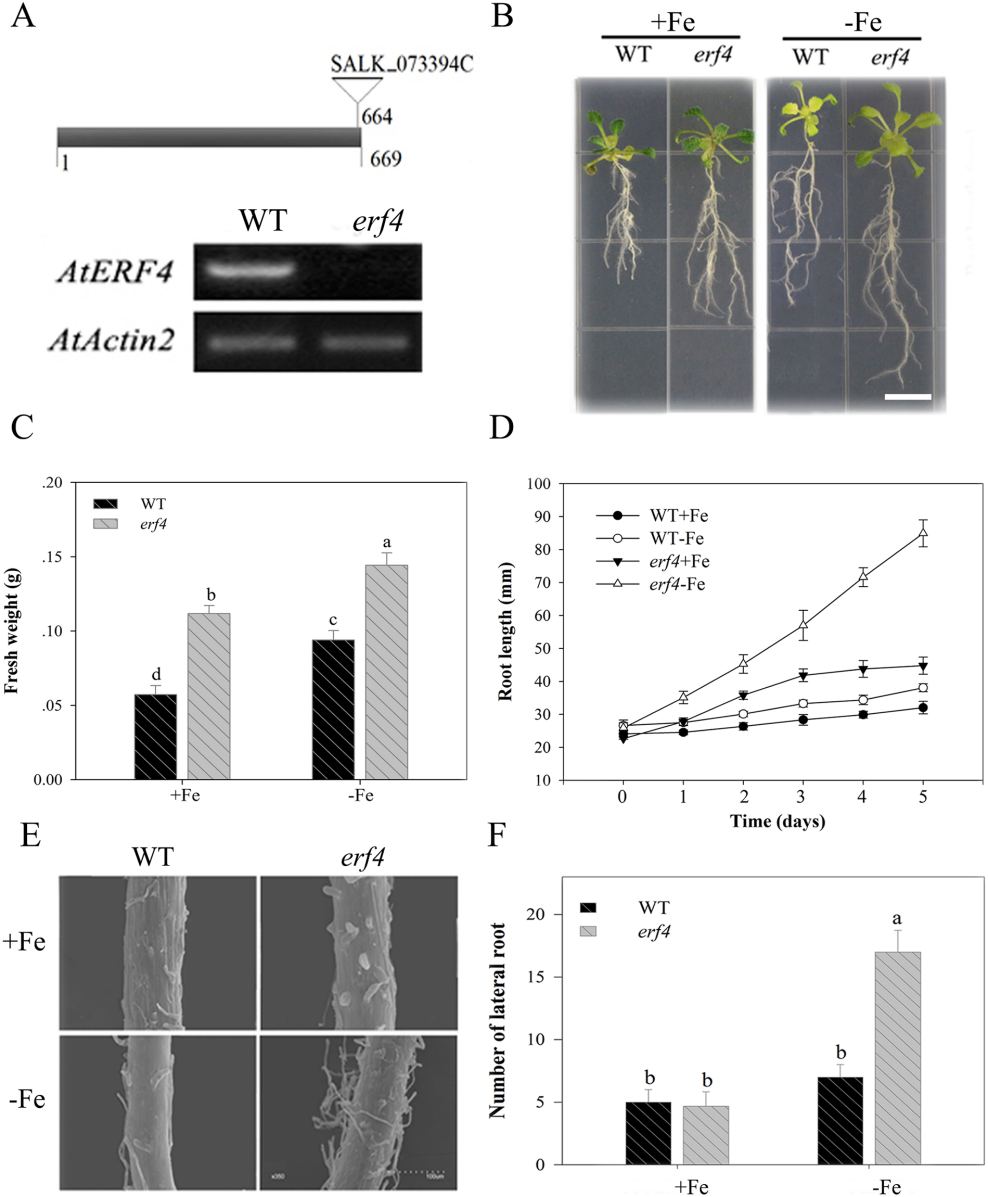
The loss of the *AtERF4* gene conducive to root development under Fe deficiency. (A) The diagram of *erf4* mutant T-DNA insertion and the expression of *AtERF4* in *erf4* mutant screened. (B) The root phenotype of the wild type (WT) and the mutant *erf4* under Fe-sufficient (100 μM EDTA-Fe) and Fe-deficient (0 μM EDTA-Fe) conditions for 5 d, scale bar = 0.8 cm. (C) The fresh weight of every five WT or *erf4* plants under Fe-sufficient and Fe-deficient conditions for 5 d. Symbols represent means ± SE of three replicates. Different letters represent statistically different means at *P* < 0.05 (one-way ANOVA with a Duncan post-hoc test). (D) The root length of WT and mutant *erf4* under Fe-sufficient and Fe deficient conditions for 5 d. Symbols represent means ± SE of three replicates. (E) The root hair quantity of WT and mutant *erf4* under Fe-sufficient and Fe-deficient conditions for 5 d was observed with a scanning electron microscope. (F) The lateral root number of WT and mutant *erf4* under Fe-sufficient and Fe-deficient conditions for 5 d. Bars represent means ± SE of three replicates. Different letters represent statistically different means at *P* < 0.05 (one-way ANOVA with a Duncan post-hoc test).

Previous research has shown that the production of ethylene induced by Fe deficiency can cause a series of signal transduction reactions involving this hormone [32]. We therefore induced *ERF4* expression in leaves and roots, and utilized the ethylene response factor *erf4* mutant to identify the role of *AtERF4* in responses to Fe deficiency. Results confirm the function of this mutant in the ethylene signaling pathway as the *erf4* (SALK_073394C) mutant contains a single T-DNA insertion site within its exon (Fig 3A) [61]. We screened this mutant using RT-PCR and showed no expression of *AtERF4* (Fig 3A). In addition, observations show that plants containing the *erf4* mutant were both bigger and apparently healthier than their wild type counterparts (Fig 3B and 3C). This result therefore implies that *ERF4* inhibits plant growth and that this effect can be reversed by promoting unlimited Fe acquisition. In addition, results show that root growth rate in the *erf4* mutant was faster than in the wild type (Fig 3B and 3D), and that the growth of root hairs was also significantly enhanced under Fe deficient conditions. We observed the root hair zone using a SEM (Fig 3E); images show that the development of root hair density and length in *erf4* plants were both much higher than in wild-type plants under Fe deficient conditions. The *erf4* mutant also had a greater number of lateral roots than its wild type counterpart (Fig 3F).

**Fig 4:**
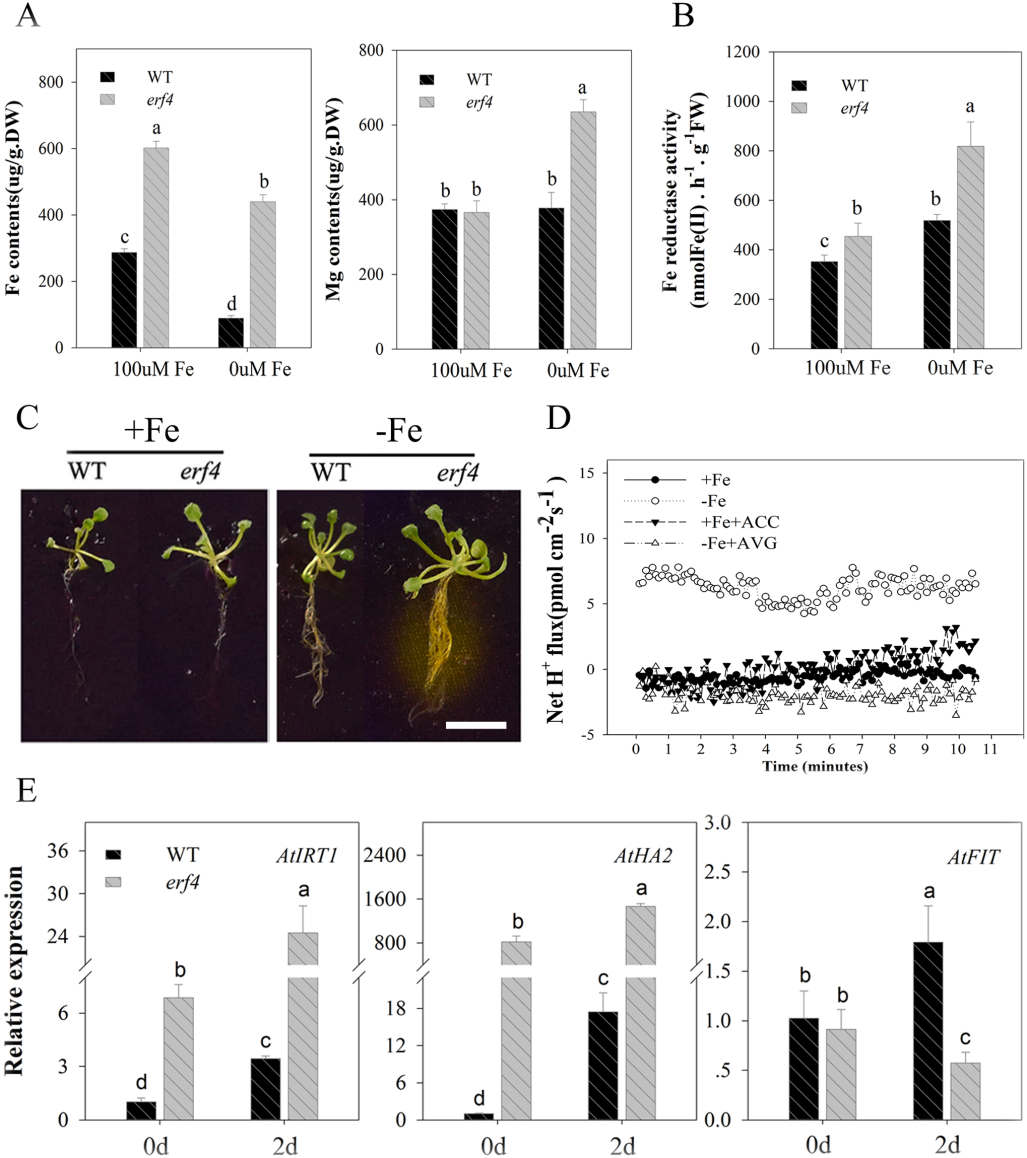
The loss of the *AtERF4* gene leads to an increased Fe deficiency response. (A) The Fe and Mg content of WT and mutant *erf4* root under Fe-sufficient and Fe-deficient for 5 d. (B) The Fe reductase activity of WT and mutant *erf4* root under Fe-sufficient and Fe-deficient conditions for 5 d. Bars represent means ± SE of three replicates. Different letters represent statistically different means at *P* < 0.05 (one-way ANOVA with a Duncan post-hoc test). (C) The qualitative analysis of the rhizosphere H_2_ of WT and mutant *erf4* under Fe-sufficient and Fe-deficient conditions for 5 d, scale bar = 0.8 cm. (D) The transient H^+^ flux of WT and mutant *erf4* root hair under Fe-sufficient and Fe-deficient conditions for 5 d. (E) Relative expression of the Fe uptake-related genes *AtIRT1*, *AtHA2* and *AtFIT* in WT and mutant *erf4* under Fe-sufficient and Fe-deficient conditions for 2 d. Bars represent means ± SE of three replicates. Different letters represent statistically different means at *P* < 0.05 (one-way ANOVA with a Duncan post-hoc test).

The elements Fe and Mg are both key factors that can alter chlorophyll content. However, the effects of ethylene on chlorophyll content via *ERF4* under Fe deficient conditions remains unclear. To evaluate this, we tested Fe and Mg content in the roots of *erf4* plants (Fig 4A); results show that loss of *AtERF4* can increase absorption of Fe in roots, and that this higher transport efficiency makes the Fe content in *erf4* mutants higher than in wild type plants under Fe deficiency. At the same time, the Mg content in *erf4* mutants was higher than in wild type plants given a Fe deficiency. This implies that an increase in *erf4* Mg content may be the result of the transportation of this element and an increase in uptake ability in *erf4* roots.

Previous work has shown that Fe uptake by plants that utilize strategy I is closely related to root ferric reductase (FCR) activity and rhizosphere pH [62, 63]. Our results show that the FCR activity of the wild type and *erf4* plants varied: for the latter, it increased with time, and the increase in Fe deficiency was significantly higher than that of the wild type plants (Fig 4B). The qualitative area of rhizosphere acidification was also larger in *erf4* plants than in wild type, and this region secreted a large amount of hydrogen (Fig 4C). Data on H^+^ flux in the root hair region (Fig 4D) show that effluxes of these ions in wild type plants occurred under Fe deficiency, and that the efflux rate of the *erf4* mutant was 2.5 times that of the wild type. These *erf4* mutants also promoted environmental acidification and thus increased the solubility of Fe^2+^. Thus, taken together, these results demonstrate that loss of *AtERF4* enhances a series of plant responses to Fe deficiency.

One of the aims of this study was to show that *AtERF4* contributes to the regulation of Fe uptake, and that the expression of Fe uptake-related genes was detected in the mutant *erf4* (Fig 4E). High expression levels of the Fe transporter gene *AtIRT1* and the H^+^-ATPase gene *AtHA2* were induced by Fe deficiency for 48 hours, while the expression of *AtFIT* was reduced following deletion of the *AtERF4* gene. Results show that *AtERF4* can regulate expression of the Fe absorption related genes *AtIRT1* and *AtHA2*; at the same time, however, there may also be interaction between *AtERF4* and other transcription factors which regulate and accumulate *AtFIT*. We therefore hypothesize that the balance between these transcription factors affects the functional genes expression under Fe deficient conditions.

### Effects of AtERF4 on chlorophyll content and the expression of chlorophyll degradation genes in *A*. *thaliana*

**Fig 5:**
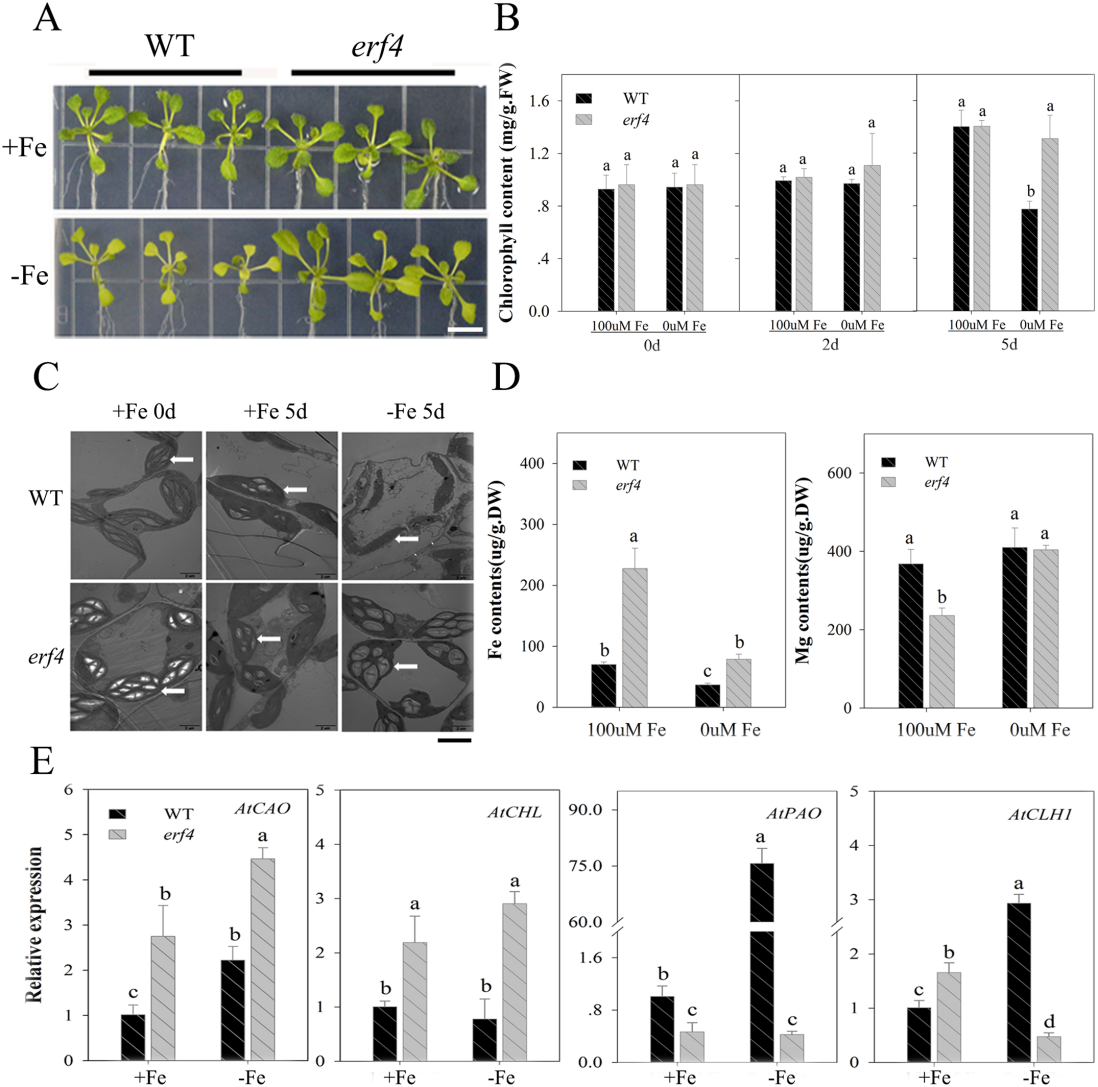
The effect of *AtERF4* on chlorophyll degradation. (A) The phenotype of the wild type (WT) and the mutant *erf4* under Fe-sufficient (100 μM EDTA-Fe) and Fe-deficient (0 μM EDTA-Fe) conditions for 5 d, scale bar = 0.8 cm. (B) The chlorophyll content of WT and mutant *erf4* under Fe-sufficient (100 μM EDTA-Fe) and Fe-deficient (0 μM EDTA-Fe) conditions for 0, 2, and 5 d. Bars represent means ± SE of three replicates. Different letters represent statistically different means at *P* < 0.05 (one-way ANOVA with a Duncan post-hoc test). (C) The chloroplast structure of WT and mutant *erf4* under Fe-sufficient (100 μM EDTA-Fe) and Fe-deficient (0 μM EDTA-Fe) conditions for 5 d. White arrows indicate the chloroplast structure state of the seedling leaves, scale bar = 3 μm. (D) The Fe and Mg content in leaves of WT and mutant *erf4* under Fe-sufficient (100 μM EDTA-Fe) and Fe-deficient (0 μM EDTA-Fe) conditions for 5 d. Bars represent means ± SE of three replicates. Different letters represent statistically different means at *P* < 0.05 (one-way ANOVA with a Duncan post-hoc test). (E) Expression of the chlorophyll biosynthesis gene (*AtCAO*, *AtCHL*) and degradation gene (*AtPAO*, *AtCLH1*) of WT and mutant *erf4* under Fe-sufficient (100 μM EDTA-Fe) and Fe-deficient (0 μM EDTA-Fe) conditions for 2 d. Bars represent means ± SE of three replicates. Different letters represent statistically different means at *P* < 0.05 (one-way ANOVA with a Duncan post-hoc test).

Results show that the loss of *AtERF4* significantly enhances the adaptability of above-ground plant sections to Fe deficiency; leaves were larger and greenness was maintained under this treatment (Fig 5A). However, the chlorophyll content of wild type leaves began to decline after two days under Fe deficient conditions and continued to fall over time. In contrast, the chlorophyll content of mutant *erf4* plants remained significantly higher than those of wild type plants under Fe deficient conditions. The chlorophyll contents of plants containing *erf4* mutants remained stable level for five days under Fe deficient conditions (Fig 5B).

We investigated the chloroplast structure of plants using TEM images. These observations show that although wild type plant chloroplast structures were damaged by Fe deficiency after five days, those of *erf4* plants remained intact (Fig 5C). Results also show that the Fe content in leaves of mutant *erf4* were higher than those of wild type plants, and the content of mutant *erf4* Mg increased more than wild type plants under iron deficiency conditions (Fig 5D). These results therefore show that *AtERF4* plays an important role in chlorophyll stabilization.

We also detected expression levels of the chlorophyll biosynthesis genes, *AtCAO* and *AtCHL*, as well as the degradation genes, *AtPAO* and *AtCLH1* (Fig 5E). Results show that expression of *AtCAO* and *AtCHL* were up-regulated in *erf4* while expression of *AtPAO* and *AtCLH1* were inhibited by Fe deficiency for two days. At the same time, enhanced expression of *AtCLH1* was also achieved in *erf4* treated with 100 μM Fe. These results suggested that the loss of *AtERF4* could alleviate the leaf-yellowing phenomenon in the early stage of Fe deficiency by regulating the related genes of chlorophyll degradation.

### *AtERF4* regulation and expression of Fe uptake-related and chlorophyll degradation-related genes

Observations show that ERF transcription factors are able to bind to GCC-box and DRE/CRT elements and that their conserved sequences are GCCGCC and CCGAC, respectively. In this GCC motif, the third C, fourth G, and last C are necessary for the ERF protein to be recognized, while all the other GCCGCC positions contribute to the selective binding of *AtERFs* [64, 65]. At the same time, some EAR motifs (^L^/_F_DLN^L^/_F_(X)P) of ERF proteins are transcriptional repressors, including AtERF4, AtERF7, AtERF10, AtERF11, and AtERF12, all known to subdue gene expression [46]. Although the EAR motif is essential for gene repression [66], the regulatory effects of ERF proteins that contain these motifs have not yet been demonstrated.

**Fig 6:**
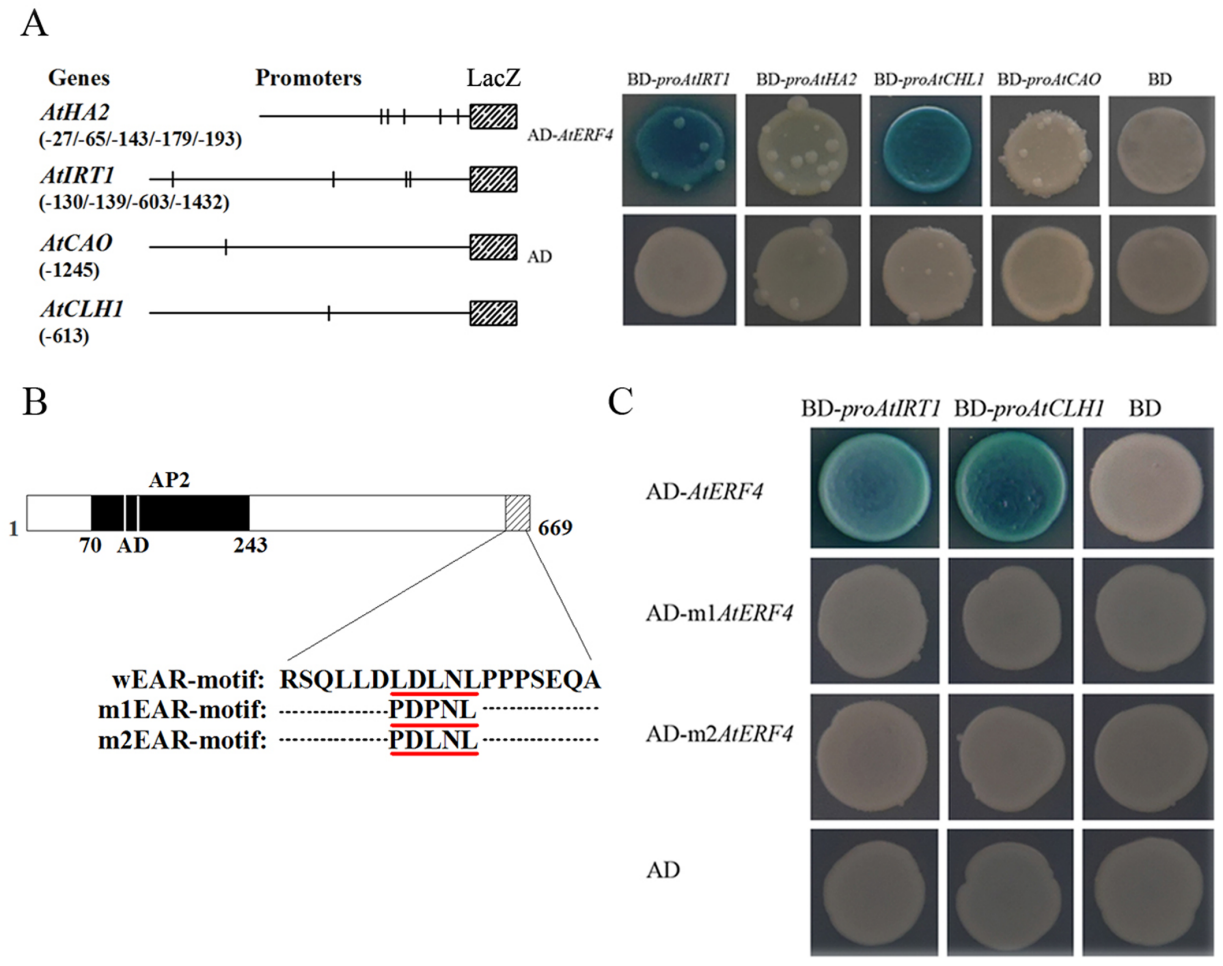
Ethylene response factor *AtERF4* bound to the promoter of *AtIRT1* and *AtCLH1* through the complete structure of EAR-motif. (A) Yeast one-hybrid showed that *AtERF4* could directly bind to the promoters of *AtIRT1* and *AtCLH1*. The structure of the *AtHA2* promoter (970 bp) revealed five GCC-box motifs (-27 bp, -65 bp, -143 bp, -179 bp and -193 bp). The structure of the *AtIRT1* promoter (1500 bp) revealed four GCC-box motifs (-130 bp, -139 bp, -603 bp and -1432 bp). There was a single GCC-box motif for the promoters (1500 bp) of *AtCAO* and *AtCLH1*, which was located at -1245 bp and -613 bp, respectively (left). All tests were conducted on media containing adenine (-Trp,-Ura). Interactions were determined based on cell growth and were confirmed by an X-gal assay on a medium lacking adenine (-Trp, -Ura) (right). (B) The coding region sequence and EAR-motif point mutation structure diagram of *AtERF4* wEAR-motif (LDLNL) mutated as m1EAR-motif (PDPNL) and m2EAR-motif (PDLNL). (C) Yeast one-hybrid demonstrated that the *AtERF4* EAR-motif site-directed mutation could not bound to the promoters of *AtIRT1* and *AtCLH1*. All tests were conducted on media containing adenine (-Trp,-Ura). Interactions were determined based on cell growth and were confirmed by an X-gal assay on a medium lacking adenine (-Trp,-Ura).

We then utilized a database of nucleotide sequence motifs that comprise plant cis-acting regulatory DNA elements (PLACE) to analyze the promoters of Fe absorption functional genes. The results of these comparisons show that between four and five ERF protein binding elements are included within the promoters of two Fe uptake-related genes, *AtHA2* and *AtIRT1*, as well as a single GCC-box motif in the promoters of two chlorophyll related genes, *AtCAO* and *AtCLH1*. In addition, the results of our yeast one-hybrid assay shows interaction between *AtERF4* and the promoters of *AtIRT1* and *AtCLH1* (Fig 6A). We therefore performed site-directed mutagenesis on the EAR region (LDLNL) of ERF4; substitution of the first and third leucine residues within LDLNL by proline (i.e. PDPNL and PDLNL) obliterated their regulatory function (Fig 6B and 6C). This result implies that the complete EAR domain structure is key to regulation, and that the presence of three leucine amino acids forms the basis of the interactions between AtERF4 and the promoter. These results therefore show that AtERF4 may be involved in regulating the expression of *AtIRT1* and thus controlling the absorption of ferrous ions, while at the same time regulating the expression of *AtCLH1* and its resultant function in leaves.

### Transient over-expression of *AtERF4* in affects chlorophyll content and Fe absorption gene expression

**Fig 7:**
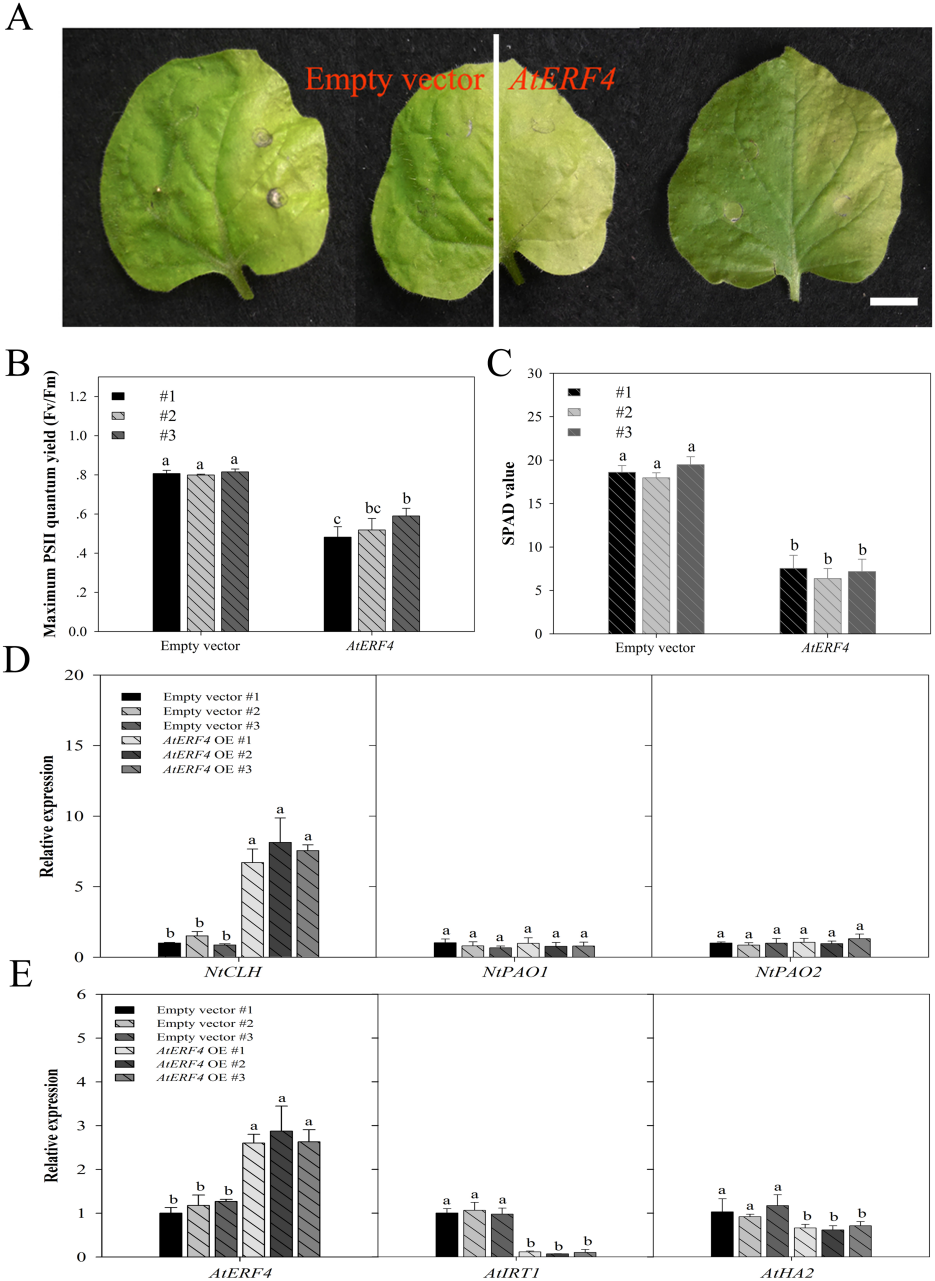
Transient over-expression of *AtERF4* promoted chlorophyll degradation and restrained Fe absorption related gene expression. (A) The phenotype of *N*. *tabacum* leaves transformed with an empty vector (left half of the blade) and over expression *AtERF4* (right half of the blade), which were carried by *Agrobacterium*, scale bar = 0.8 cm. (B, C) The maximum PSII quantum yield (Fv/Fm) and the chlorophyll content (SPAD value) were analyzed, respectively, using a photosynthesis yield analyzer (MINI-PAM-II, Walz, Germany) and a portable chlorophyll meter (SPAD-502 Plus; Konica Minolta Inc, Japan). Bars represent means ± SE of three replicates. Different letters represent statistically different means at *P* < 0.05 (one-way ANOVA with a Duncan post-hoc test). (D) Transient over-expression (OE) of *AtERF4* influenced the expression of chlorophyll degradation genes *NtCLH* in *N*. *tabacum* leaves. Bars represent means ± SE of three replicates. Different letters represent statistically different means at P < 0.05 (one-way ANOVA with a Duncan post-hoc test). (E) Transient over-expression (OE) of *AtERF4* influenced the expression of Fe transporter gene *AtIRT1* in *A*. *thaliana* roots. Bars represent means ± SE of three replicates. Different letters represent statistically different means at P < 0.05 (one-way ANOVA with a Duncan post-hoc test).

Our results show that transient over-expression of *AtERF4* in *Nicotiana tabacum* causes chlorosis symptoms in right leaf blades compared to empty vectors (i.e. left leaf blades) (Fig 7A). At the same time, results show that maximum PSII quantum (Fv/Fm) (between 0.4817 and 0.5900) (Fig 7B) and chlorophyll content (SPAD) (between 6.3667 and 7.5333) (Fig 7C) were both significantly lower in the right compared to left leaf blades. Values for Fv/Fm and SPAD for empty vectors were between 0.7993 and 0.815 and between 17.9667 and 19.4667, respectively, while over-expression of *AtERF4* triggered the expression of *NtCLH* (Fig 7D). In contrast, expression of the chlorophyll degradation genes *NtPAO1* and *NtPAO2* was similar between over-expressed *AtERF4* plants and empty vectors (Fig 7D). We also find that transient over-expression (OE) of *AtERF4* in *A*. *thaliana* root leads to significant downregulation of the expression of Fe uptake genes *AtIRT1* compared with empty vector plants. But the expression of *AtHA2* gene in OE plants changed little (Fig 7E).

## Discussion

The hormone ethylene is produced in various higher plant organs; Fe deficiencies can induce ethylene production in plants using strategy I as well as in the roots of rice. Thus, ethylene is an important signaling substance in processes related to Fe deficiency stress, and plays a positive role in regulating plant responses to Fe deficiency [23, 24, 28, 34].

Surprisingly few studies have addressed the role of ERF in plant responses to Fe deficiency [47]. In one exception, Wang et al. [32] recently showed that the expression of ERF transcription factors (e.g. MxERF4 and MxERF105) in apple rootstock (*Malus xiaojinensis*) induced up-regulation under Fe deficient conditions. It is well-known that ERF lies downstream with respect to the ethylene pathway [67]; this hormone initiates ERF1 activity by activating the EIN3/EIL1 transcription factor that forms part of signal transduction and response. It is also possible that some ERFs may regulate ethylene synthesis [25]. AtERF4 is a transcriptional repressor capable of modulating ethylene and abscisic acid responses [46]. Some research reported that *ERF4* expression can affect drought, salt and Fe stress in plants [32, 47, 49]. However, the function of ERF4 in the control of responses to low Fe levels and in the chlorophyll degradation of strategy I plants has not yet been documented.

Our results demonstrate that *AtERF4* is highly expressed in *A*. *thaliana* roots and leaves. The ethylene produced when these plants experience low levels of Fe promotes the expression of a series of transcription factors, while degradation of chlorophyll in the leaves of wild-type *A*. *thaliana* is alleviated in the presence of the ethylene synthesis precursor ACC. However, the chlorophyll content of the *erf4* mutant was higher in our study, which implies that there should be a relationship between ethylene content and *AtERF4* in chlorophyll degradation pathway regulation. The results of both our yeast experiment and transiently over-expressed *AtERF4* in tobacco leaves confirmed that ERF4 regulates the expression of the chlorophyll degradation gene, *CLH1*. This transcription factor boosts *CLH1* up-regulated expression as much as possible to maintain leaf chlorophyll content for a certain period of time, while the continued accumulation of ethylene eventually leads to senescence stress in plants, manifested by symptoms of chlorosis [68]. This result therefore suggests that ERFs are important transcription factors in the ethylene response pathway.

Recent research has also shown that Fe deficiency leads to the enhanced release of ethylene by *A*. *thaliana*; this is because *MPK3/MPK6* regulate activity of the ethylene synthesis gene *ACS2/ACS6* to induce the production of this hormone [58]. Garcia et al. [21] showed that the transcription abundance of *AtACS6* increases after just one day at low Fe levels. Consistent with previous work, expression of *ACS2/ACS6* in the roots and leavse of *A*. *thaliana* was induced after two days of Fe deficient conditions in this study. We also detected the expression of genes that respond to Fe deficiency in roots and demonstrate found that *AtERF4* regulates the Fe uptake-related gene *AtIRT1*. Surprisingly, however, *AtERF4* does not bind to the promoter of *AtHA2* (Fig 5), although transcription of the latter changed in mutant *erf4*; thus, the expression of *AtHA2* may be regulated by another protein which is able to interact with AtERF4. At the same time, *FIT* expression under Fe deficiency in *erf4* mutant is reduced but FCR activity in mutant *erf4* roots is higher than that of WT plant roots. We hypothesize that there may be other transcription factor upregulating *FIT* expression in the absence of Fe. It is therefore possible that the balance between these transcription factors affects the function of genes expressed because of Fe deficiency. Our experiments show that the content of Fe and Mg in *erf4* mutant leaves and roots increased; this result indicates that AtERF4 also plays a role in the absorption and transport of Fe and two-valence cations. The ethylene signal therefore provides a mechanism to positively regulate Fe absorption, mainly through via the ethylene-core transcription factor EIN3/EIL1 which interacts with the FIT protein; previous work has show that stable FIT protein levels increase plant Fe absorption in environments where this element is scarce [34]. The results of both our yeast one-hybrid assay and transiently over-expressed *AtERF4* in *A*. *thaliana* roots demonstrated that ERF4 negatively regulate Fe deficiency responses; it is therefore the case that the balance function of Fe uptake in plants is affected by *AtIRT1*, itself regulated in expression by different transcription factors.

A large number of root hairs is one of the most important morphological features that characterizes responses to Fe deficiency; the formation of adventitious roots enables plants growing under Fe deficient conditions to absorb enough Fe element to maintain a normal metabolic level [69]. The results of this study also show that ethylene plays a role in root hair induction under Fe deficient conditions; the number of root hairs in *erf4* mutant increased compared wild type plants that were not treated with Fe supplements. Root growth rate in these mutant was also faster than in wild type plants. Our results suggest that ERF4 may play an important role in regulating root system changes and hair abundance in plants as they respond to Fe deficiencies and may therefore suggest fruitful avenues for future research.

**Fig 8:**
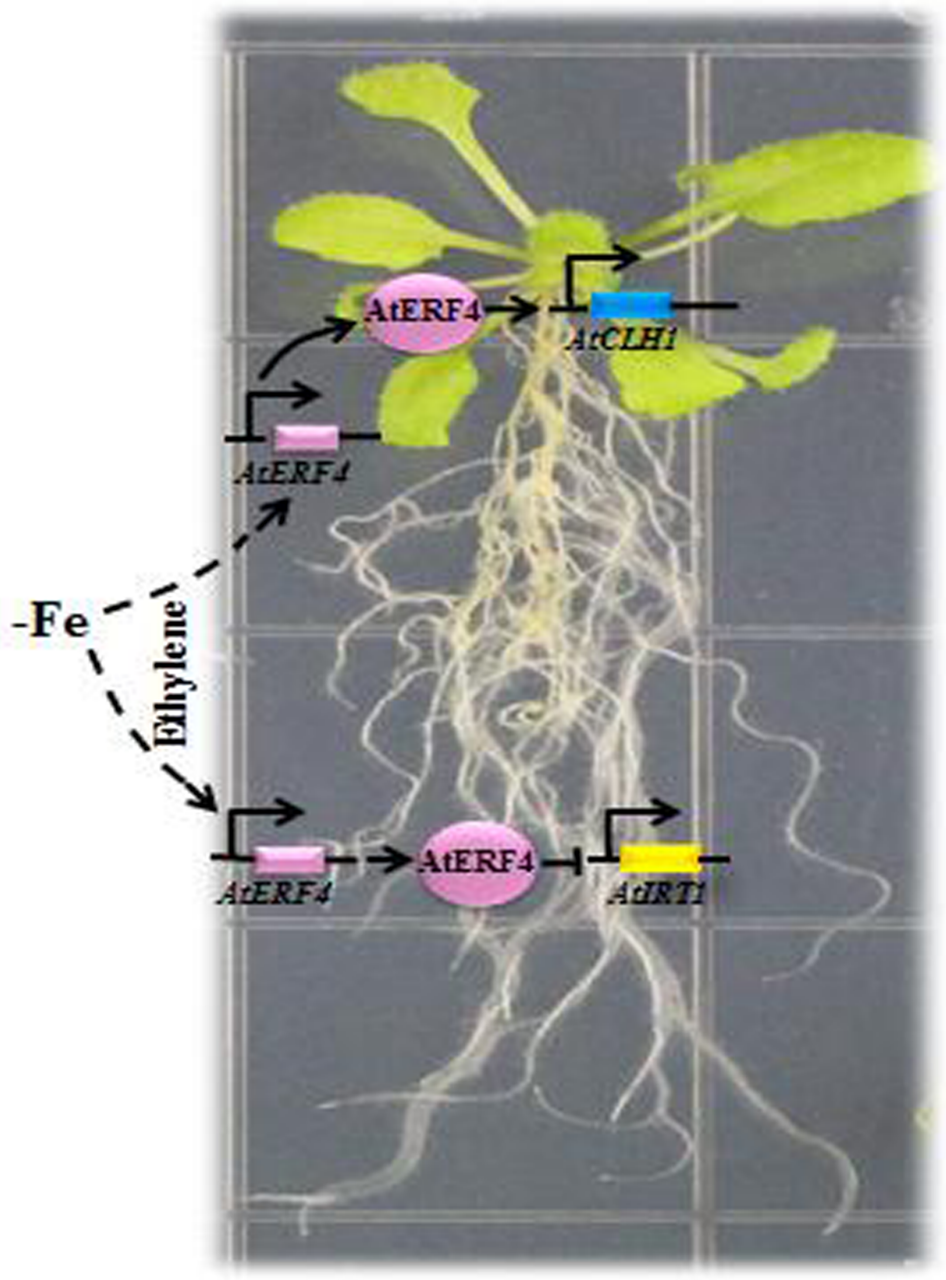
Model of the AtERF4-mediated Fe deficiency responses. The early stages of Fe deficiency led to ethylene production and induced the expression of the ethylene response factor AtERF4 in the root and leaf parts. Then AtERF4, as an important transcription factor, negatively regulated expression of the Fe transporter (AtIRT1) by directly binding to the promoter of *AtIRT1* in *A*. *thaliana* root to affect the uptake of Fe and Mg. In addition to its role as a transcription factor, AtERF4 could directly regulate the expression of the chlorophyll degradation gene *AtCLH1*, and thus cause the chlorophyll content of a leaf to decrease, leading to leaf chlorosis under conditions of Fe deficiency.

In conclusion, the ethylene biosynthesis genes (*AtACS2* and *AtACS6*) and ethylene response factor gene *AtERF4* were expressed in *A*. *thaliana* roots and leaves resulted from early stage Fe deficiency. Our results show that AtERF4 is an important factor involved in plant responses to Fe deficiency in the downstream ethylene pathway; we have confirmed this via a series of processes, phenotypic observations, and yeast validation tests. Thus, *AtERF4* could be transcribed in the early stage of Fe deficiency, as it participates in the negative regulation of the gene *AtIRT1* in the root system and promotes the expression of the chloroplast degradation gene *AtCLH1* (Fig 8). We have shown that AtERF4 regulates the expression of *AtIRT1* and *AtCLH1* and therefore influences responses to Fe levels in plants.

## Supporting information

S1 Table Primer sequences used in this study. (DOCX)
