# Supplementary material for: The ethylene response factor AtERF4 negatively regulates the iron deficiency response in Arabidopsis thaliana
Source: PLoS One. 2017 Oct 18;12(10):e0186580. doi: 10.1371/journal.pone.0186580 (PMC5646859; doi:10.1371/journal.pone.0186580)
Supplement: S1 Table — (DOCX) [file pone.0186580.s001.docx]

**S1** **Table Primer sequences used in this study.**

| **Gene**  **name** | **ID** | **Sequence (5’-3’)** | **Used for** |
| --- | --- | --- | --- |
|  |  |  |  |
| AtERF4 | SALK_073394C LP | AAAGGAGCGTCGCTTTTACTC | Genotyping *ERF4*  WT allele |
|  | SALK_073394C RP | AAATTTCGGAAATTGGATTGG |  |
|  | SALK_073394C RP | AAATTTCGGAAATTGGATTGG | Genotyping *erf4*  mutant allele |
|  | SALK LBb1.3 | ATTTTGCCGATTTCGGAAC |  |
| AtERF4 | AtERF4-F1 | ATGGCCAAGATGGGCTTGAA | Gene amplification |
|  | AtERF4-R1 | TCAGGCCTGTTCCGATGGAG |  |
|  | AtERF4-F2 | CGGAATTC ATGGCCAAGATGGGCTTGAA | Yeast one-hybrid |
|  | AtERF4-R2 | CCGCTCGAG TCAGGCCTGTTCCGATGGAG |  |
|  | AtERF4-F3 | GCTCTAGAATGGCCAAGATGGGCTTGAA | Transient over-expression |
|  | AtERF4-R3 | CGGGATCCGGCCTGTTCCGATGGAGGAG |  |
| AtIRT1 | proAtITR1-F | GCTCTAGA ATTAAAAAATATTTCTTTTCTC | Promoter amplification |
|  | proAtITR1-R | CCGCTCGAG TCTTAAATTTGCATATTATT |  |
| AtHA2 | proAt HA2-F | CGGAATTCGTATGTAGTTTTTGTTATAA | Promoter amplification |
|  | proAt HA2-R | CCGCTCGAGCTCTCACCACTCCTTCACTG |  |
| AtCLH1 | proAtCLH1-F | CCCAAGCTTCAAAAAATGAAATGTCTCTCGATCTA | Promoter amplification |
|  | proAtCLH1-R | CCGCTCGAGTTGTATTAAACTAGATAATTAAA |  |
| AtCAO | proAtCAO-F | CGGAATTCCCCGAGAAGAGAGAGCTTCG | Promoter amplification |
|  | proAtCAO-R | CCGCTCGAGGGCGTCACCGGAAAGAGAGT |  |
|  | AtACS2-F | TACCACGGGGATCAAGAAAG | qRT-PCR |
|  | AtACS2-R | AGGAAGAGCCAGGAGACACA |  |
|  | AtACS6-F | TGGTTGGTTAAAGGCCAAAG | qRT-PCR |
|  | AtACS6-R | CTCTAAACCATCCCGGTTCA |  |
|  | AtERF4-F4 | GACTGTGCTTCTCCTCCGAC | qRT-PCR |
|  | AtERF4-R4 | CGACCTACGTTACCGATCCC |  |
|  | AtCAO-F | CTGGGATCCATATCCAATCG | qRT-PCR |
|  | AtCAO-R | CAGCGAAATGTCTCCACAGA |  |
|  | AtCHL-F | GGGAAAGTTCAATGCGTGGG | qRT-PCR |
|  | AtCHL-R | CCTGGATTTGGCGTCCTTGA |  |
|  | AtPAO-F | CAGCTTGGTGGCAGGTTGTA |  |
|  | AtPAO-R | GAGGTTGGTTAGACGGGGTG |  |
|  | AtCLH1-F | TTCTTGTAGCCCCACAGTTG |  |
|  | AtCLH1-R | GCCCACGAGTGAGGTGTATT |  |
|  | AtFIT-F | CCATTGTTGGAGATGCAGTG | qRT-PCR |
|  | AtFIT-R | TACCGCGAAAAGGTTGAGTC |  |
|  | AtHA2-F | AGAGATGGCAGGAATGGATG | qRT-PCR |
|  | AtHA2-R | ATGGCATCCTGGTTCTCAAC |  |
|  | AtIRT1-F | CTCCAACCAGACGGAAACAT | qRT-PCR |
|  | AtIRT1-R | TACCAACTGCGTTCTTGCTG |  |
|  | AtActin2-F | GGTAACATTGTGCTCAGTGGTGG | qRT-PCR |
|  | AtActin2-R | AACGACCTTAATCTTCATGCTGC |  |
|  | NtCLH-F | GGCTAGCTGAAGGATTGCAC | qRT-PCR |
|  | NtCLH-R | ATAGGTGAGAACCGCTGGTG |  |
|  | NtPAO1-F | TGCGTGTTATCTTGGCTTCA | qRT-PCR |
|  | NtPAO1-R | GGGTGTCGAGATTAGCAGGA |  |
|  | NtPAO2-F | CTCCACGAACTGCAGAAGAA | qRT-PCR |
|  | NtPAO2-R | GTTTAGGGATGTCGAGATTGG |  |
|  | NtActin-F | GGGTTTGCTGGAGATGATGCT | qRT-PCR |
|  | NtActin-R | GCTTCGTCACCAACATATGCAT |  |
